# Reverse zoonosis of the 2022–2023 human seasonal H3N2 detected in swine

**DOI:** 10.1038/s44298-024-00042-4

**Published:** 2024-08-13

**Authors:** Michael A. Zeller, Daniel Carnevale de Almeida Moraes, Giovana Ciacci Zanella, Carine K. Souza, Tavis K. Anderson, Amy L. Baker, Phillip C. Gauger

**Affiliations:** 1 Department of Veterinary Diagnostic & Production Animal Medicine, Iowa State University, Ames, IA 50011-1134 USA; 2 Programme in Emerging Infectious Diseases, Duke-NUS Medical School, Singapore, 169857 Singapore; 3 Virus and Prion Research Unit, National Animal Disease Center, USDA-ARS, Ames, IA 50010 USA; 4 Department of Veterinary Microbiology and Preventive Medicine, Iowa State University, Ames, IA 50011-1134 USA

**Keywords:** Influenza virus, Viral epidemiology, Viral evolution, Viral transmission

## Abstract

The Iowa State University Veterinary Diagnostic Laboratory detected nineteen human-to-swine reverse zoonoses of the 2022–2023 human seasonal H3N2 between November 2022 and November 2023. Cases from seven U.S. locations were detected: 3 Colorado, 1 Illinois, 1 Indiana, 2 Missouri, 7 North Carolina, 1 Ohio, and 1 Pennsylvania. One additional case was detected in Mexico and two cases were identified from Chile. Case samples were comprised of 4 nasal swabs and 15 oral fluids. Virus was successfully isolated from two of four nasal swab samples, but isolation from oral fluids was unsuccessful. The swine detections of H3 human viruses were classified to one of two human-seasonal H3 clades, 3C.2a1b.2a.2b and 3C.2a1b.2a.2a.1. Phylogenetic inference indicated at minimum 7 reverse zoonotic events occurred, with possible swine-to-swine transmission following the initial spillover. Twelve neuraminidase genes were sequenced, and nine were classified as human-seasonal H3N2 lineage: the remaining were endemic swine IAV NA genes from the N2.2002B, N2.1998, or the N1.Classical lineage, suggesting reassortment. The two viral isolates obtained from nasal swab samples were sequenced and were entirely human-lineage viruses. Seven swine samples with human seasonal H3 were sequenced and revealed co-detections with H1 1A.3.3.3 (gamma), with internal gene segments from both the triple reassortant internal gene (TRIG) and pandemic 2009 lineages. Serologic investigation of samples from swine production systems provided evidence for infection with human seasonal H3N2. One farm in the United States and four farms in Mexico had concurrent virologic evidence. The swine-isolated 3C.2a1b.2a.2b H3N2 was antigenically distinct from endemic 1990.4.A, 2010.1, and 2010.2 swine H3N2 lineages, but retained antigenic similarity to a recent human seasonal H3N2 (A/Darwin/6/2021). Pigs experimentally inoculated with a representative isolate demonstrated replication in the nose and lungs and minimal to mild macroscopic and microscopic lung lesions, but primary pigs did not transmit the virus to indirect contacts. If sustained in the pig population, this human seasonal H3 would represent the first new lineage detected in pigs the 2020 decade and present an emerging threat to swine health and production.

## Introduction

H3 subtype influenza A virus (IAV) is endemic in both swine and human populations. Reverse zoonotic spillover of H3 subtype IAV from humans to swine occurred with regular frequency in the past^1–5^. The introduction of genetically distinct human lineage H3 IAV into swine has increased genetic diversity circulating within the swine population, complicating control efforts. Swine have also been the source of variant virus infections in humans^6,7^, highlighting the role and risk of inter-species transmission for the continued diversification of IAV.

Historically, there has been evidence of H3 infection in North American swine since 1980 based on serological detection^8^. However, the first isolation of H3N2 from North American swine was described in Quebec, Canada in 1990^9,10^. Between 1995–1998, H3, composed of a triple reassortant virus with segments from human, swine, and avian origins was introduced through three distinct human-to-swine spillover events denoted as Clusters I, II, and III^3,4^. Each spillover expressed moderately different antigenic properties^11^. The Cluster IV lineage evolved from Cluster III, and when first reported in 2005, had acquired a distinct human N2^12^. This lineage of virus diversified into multiple genetic clades over the last two decades, including Cluster IVA (now designated as 1990.4.a in the global swine H3 nomenclature), a major H3 lineage regularly detected in swine^13^. The presence of H3 in swine remained stable until the 2010 decade, when two H3 reverse zoonosis events were successfully established within the US swine population, denoted as 2010.1 and 2010.2^2,5,14,15^, each introducing genetically and antigenically diverse IAV into the swine population. At present, the 1990.4a and the 2010.1 lineages compose the majority of H3 detections in US swine^13,16^.

Not all detected H3 reverse zoonotic events become endemically established in swine^17^. Through the efforts of the United States Department of Agriculture’s (USDA) swine IAV passive surveillance system and the voluntary participation of producers and veterinarians, approximately 15 dead-end reverse zoonosis events were detected in swine from 2013 to 2019^16^. There were not detections of human-origin reverse zoonoses events in the swine population between 2019–2022. Notably, this occurred during the SARS-CoV-2 pandemic, when human influenza cases were reduced^18,19^. Social distancing measures put in place by governments and employers may have acted as an additional layer of biosecurity between humans and animals. With the relaxation and removal of prior SARS-CoV-2 restrictions, the number of human IAV cases in the 2022–2023 human IAV season dramatically increased. In late February of 2023, approximately 75% of the IAV positive tests reported to the CDC by U.S. public health laboratories were H3 subtype, with the majority of cases belonging to the 3C.2a1b subclade 2a.2b^20^. By early July of 2023, H3 cases dropped to approximately 6% of weekly detections but accounted for 71% of the cumulative human Influenza A and Influenza B detections in the 2022–2023 human influenza season^21^.

Historical cases have shown that reverse zoonosis introduces additional genetic and antigenically diverse IAV in swine, which complicates vaccine control efforts. Herein, we describe 19 H3 subtype IAV reverse-zoonosis events in swine in the U.S., Chile, and Mexico, occurring between November 2022 and November 2023. All swine detections were genetically related to the prevalent H3 3C.2a1b.2a.2 circulating in humans but were derived from two different subclades. The potential for these H3 introductions to become endemic within U.S. and global swine populations is an outstanding question. However, these events emphasize how human seasonal IAV is a risk to swine health and impacts the ecology and evolution of IAV in the swine host.

## Methods

### Ethics statement

All animal experiments were conducted in a manner that complies with the Code of Federal Regulations for animal welfare^22^. Ferrets were cared for in compliance with the Institutional Animal Care and Use Committee of the National Animal Disease Center, USDA, ARS. All groups of swine for airborne transmission studies were housed separately in biosafety level 2 containment rooms and cared for in compliance with the Institutional Animal Care and Use Committee of the National Animal Disease Center.

### Detection and validation of diagnostic submissions

Viral RNA extraction, subtyping, and Sanger sequencing were performed at the Iowa State University Veterinary Diagnostic Laboratory (ISU VDL) according to standard operating procedures (SOP)^1^. Briefly, viral RNA was extracted from both oral fluid and nasal swab samples under the manufacturer’s instructions (MagMAX pathogen RNA/DNA isolation kit, Thermo Fisher Scientific). Nasal swabs are specific to an individual animal whereas oral fluids are a population sample type, typically pooled from multiple swine chewing on a rope. IAV RNA was detected through reverse transcription real-time PCR (RT-rtPCR) using a commercial kit according to the manufacturer’s instructions (VetMAX Gold SIV Detection Kit, Thermo Fisher Scientific), using VetMAX Xeno as a positive internal control. The hemagglutinin (HA) and neuraminidase (NA) were subtyped from RT-rtPCR positive samples (VetMAX Gold SIV subtyping kit, Thermo Fisher Scientific), using separate reactions for H1, H3 and N1, N2. Sanger sequencing was conducted on genomic material that was amplified using a separate RT-PCR according to the manufacturer’s instructions (qScript XLT 1-step RT-PCR kit; Quantabio), using primers proprietary to the ISU VDL. Amplified genomic material was cleaned and submitted to the Iowa State University DNA facility (Ames, IA). Sequence assembly was performed using Geneious Prime^23^. Virus isolates were successfully obtained from two nasal swabs using a prior published isolation protocol^1^.

The cases in this study originated from routine submissions of clinical pigs where the client was seeking a diagnosis of the etiological agent afflicting their swine or were submitted for routine surveillance from healthy populations of swine. Additional samples were requested from a farm in North Carolina to identify continued circulation of human-like virus, yielding sample A02758610. The predominant sample type submitted was oral fluid, an antemortem pooled population sample type that is frequently used due to ease of collection.

**Fig. 1:**
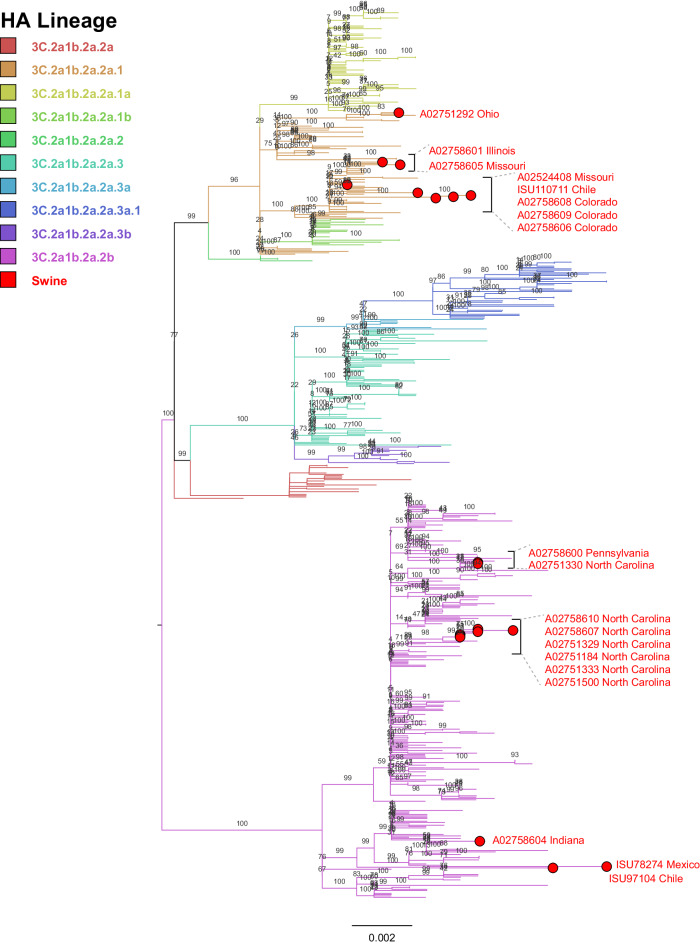
H3 3C.2a1b.2a hemagglutinin maximum-likelihood tree. Branches are colored by lineage, with swine sequences indicated by red dots and text. Each branch is labeled with bootstrap support, calculated from 1000 ultrafast bootstraps.

The index case HA sequence, A02751184 detected on November 7, 2022 in North Carolina, was automatically identified as novel by the *ISU FLU*ture automated clade designation system^16,24^. BLAST and maximum-likelihood phylogenetic analysis confirmed the virus was similar to human seasonal H3 influenza. Subsequent cases were detected and assigned a “human-to-swine-2022” designation by the automated classification system. Human H3 subtype HA sequences from the US (United States) from October 2020 to May 2023 were downloaded from GISAID on May 9th, 2023 (*n* = 13,656)^25^ and annotated with the human clade designation using Nextclade (Reference: A/Darwin/6/2021, Updated: 2023-04-02)^26^. Human sequences were paraphyletically subsampled to approximately 600 sequences, or 5% of the data using smot^27^, and aligned using MAFFT^28^. Amino acid alignments of the swine-origin sequences were visualized using Geneious Prime (Supplementary Fig. 1). An additional 47 sequences from GISAID sampled between May 9th, 2023 to December 23rd, 2023 were appended to the dataset with subsequent realignment. The clade assignment of swine gene sequences was confirmed for each virus by maximum-likelihood phylogenetic analysis using IQ-TREE, with a general time-reversible model using gamma-distributed rates and invariant sites, run with 1000 ultrafast bootstraps for branch support (Fig. 1)^29^. The antigenic motif, six amino acid sites located at 145, 155, 156, 158, 159, and 189 that have been noted to have a disproportionate effect on the antigenic phenotype, were identified for all swine sequences and genetically similar human sequences^30^. Glycosylation sites on the HA gene were predicted using NetNGlyc v1.0, using a threshold of 6/9 jury agreement and a potential greater than 50%^31^.

**Fig. 2:**
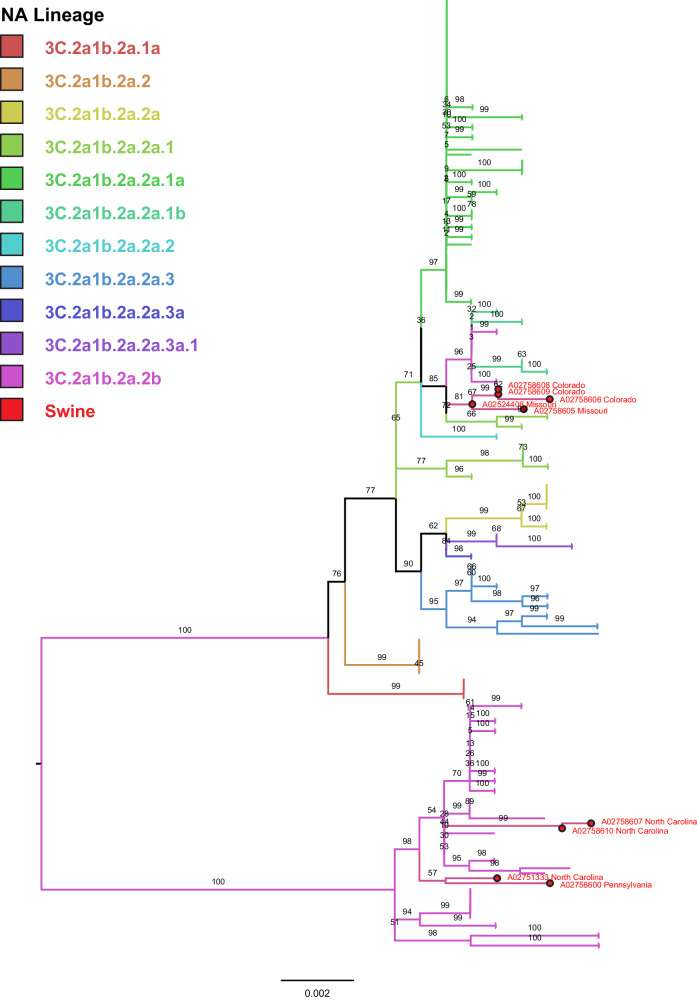
N2 human seasonal neuraminidase maximum-likelihood tree. Branches are colored by the lineage of the paired hemagglutinin, with swine sequences indicated by red dots and text. Each branch is labeled with bootstrap support, calculated from 1000 ultrafast bootstraps.

Neuraminidase sequences were obtained from 11 samples. Human-origin N2 sequences were confirmed by BLAST comparison and phylogenetic inference. A subsample of N2 neuraminidase sequences associated with prior sampled human HA sequences was aligned with swine N2 sequences using MAFFT^28^. Alignments of the swine-origin sequences were visualized using Geneious Prime (Supplementary Fig. 2). Maximum-likelihood phylogenetic trees were inferred using IQ-TREE, with a general time-reversible model using gamma-distributed rates and invariant sites, run with 1000 ultrafast bootstraps for branch support (Fig. 2).

### Whole genome sequencing of influenza A virus strains

Whole genome sequencing (WGS) was performed on seven clinical samples. Next-generation sequencing was performed on extracted viral RNA (MiSeq platform, Illumina) following standard Illumina protocols at the ISU VDL using TruSeq sequencing libraries (TruSeq stranded total RNA library preparation kit, catalog 20020596, Illumina). Reads were trimmed and assembled based on prior published ISU VDL protocols^1^. Assembled sequences were classified into genetic clades or evolutionary lineage using octoFLU^32^.

### Temporal reconstruction of IAV transmission in the United States

A low parameter Bayesian model was employed to infer a temporal phylogenetic tree using BEAST version 1.10.4. The analysis used host as a trait (19 swine, 617 human), and incorporated an asymmetric substitution model using BSSVS to infer the social network^33–39^. The model implemented an uncorrelated relaxed clock and had state change counts reconstructed with change history logged to the tree, an HKY85 substitution model, and an exponential growth tree prior, and was run for 200,000,000 states logging every 10,000 states. Maximum clade credibility (MCC) trees were built using 20% burn-in using the Tree Annotator program of the BEAST 1.10.4 package. Subtrees inclusive of the swine sequences from North Carolina and Colorado were directly sampled from the MCC tree for visualization.

Human seasonal influenza virus data was downloaded from CDC FluView interactive on April 10, 2023, for influenza seasons 2009–2010 to 2022–2023. The number of H3 positive detections was divided by the total positive cases per season to determine the percentage of H3 positives per year. The linear relationship between the percentage of H3 detections per season and number of observed H3 human-to-swine spillovers was assessed using the Pearson correlation coefficient.

### Serologic detections

Serologic testing indicated additional introduction events of human-origin H3N2 in commercial swine. Five commercial breeding farms (A–E) were NP ELISA positive for IAV (Swine Influenza Virus Antibody Test, IDEXX, Westbrook, ME) during the influenza season of 2022–2023. These farms were different than the farms that submitted samples for sequencing. All but one had evidence of IAV detection in oral fluids. Serum samples obtained from these five farms were tested against a panel of human and swine IAV by hemagglutination inhibition (HI) assays. Sera were treated by heat inactivation at 56 °C for 30 min, 20% kaolin in PBS incubation for 20 min and adsorbed with 0.5% turkey red blood cells for 20 min to remove nonspecific hemagglutination inhibitors and natural serum agglutinins. HI assays were performed using standard techniques^40^, using PBS as a negative control in place of virus. Group HI titers were transformed into log_2_ geometric means for comparison purposes. The HI antigens represented human seasonal clade 3C.2a1b.2a.2a.1 (A/Darwin/6/2021) and a human-origin swine virus isolated from this investigation from farm D to represent human seasonal clade 3C.2a1b.2a.2b (A/swine/North Carolina/A02751333/2022). The additional antigens tested were a human seasonal H1N1 (A/Hawaii/70/2019-like) and representative swine H3N2 strains from lineages 1990.4.a (A/swine/Iowa/A02750897/2022), 2010.1 (A/swine/Iowa/A02636454/2022) and 2010.2 (A/swine/Indiana/A02635878/2021) to rule out co-circulation of other IAV subtypes and H3 lineages.

### Antigenic characterization

Human seasonal H3N2 vaccine strains (A/Iowa/60/2018, A/Hong Kong/45/2019) and candidate vaccine viruses (CVV)s (A/ Minnesota/11/2010 X 203, A/Ohio/13/2017-IDCDC-RG60 and A/Ohio/28/2016-IDCDC-RG55C) and antisera produced in ferret against these strains were provided by the Virology, Surveillance, and Diagnosis Branch, Influenza Division, Centers for Disease Control and Prevention (CDC), Atlanta, GA. Human seasonal H3N2 vaccine strains (A/Cambodia/E826360/2020-cell, A/Darwin/9/2021, and A/Darwin/6/2021-like) were provided by the University of Pennsylvania. Swine H3N2 isolates representative of 1990.4.a clade (A/swine/North Carolina/A02245294/2019), 2010.1 clade (A/swine/Kansas/A02245675/2020, A/swine/Utah/A02524953/2020, A/swine/Iowa/A02636454/2022A/swine/Iowa/A02636476/2022) and 2010.2 clade (A/swine/Iowa/A02524572/2020, and A/swine/Indiana/A02635878/2021) were provided by the National Veterinary Services Laboratories (NVSL) through the U.S. Department of Agriculture (USDA) IAV in swine surveillance system in conjunction with the USDA-National Animal Health Laboratory Network (NAHLN). Viruses were propagated in Madin-Darby canine kidney (MDCK) cells grown in Opti-MEM (Life Technologies, Waltham, MA). The virus growth media contained antibiotics and antimycotics and 1 μg/ml of tosylsulfonyl phenylalanyl chloromethyl ketone (TPCK)-trypsin (Worthington Biochemical Corp., Lakewood, NJ).

H3N2 antisera was produced in ferrets against human seasonal strains (A/Cambodia/E826360/2020-cell, and A/Darwin/6/2021-like), and representative swine strains of clades: 1990.4.a (A/swine/North Carolina/A02245294/2019), 2010.1 (A/swine/Utah/A02524953/2020) and 2010.2 (A/swine/Iowa/A02524572/2020) by intranasal inoculation with 10^6^ TCID_50_/ml of each strain. Blood was collected when ferrets reached an HI titer of >160. Ferrets were cared for in compliance with the Institutional Animal Care and Use Committee of the National Animal Disease Center, USDA, ARS.

Prior to HI assays, ferret antisera were heat inactivated at 56 °C for 30 min, then treated with a 20% Kaolin suspension (Sigma-Aldrich, St. Louis, MO), followed by an adsorption with 0.75% guinea pig red blood cells to remove nonspecific agglutinins and inhibitors of hemagglutination. HI assays were performed using 20 nM of oseltamivir carboxylate^41^, using PBS as a negative control in place of virus. Cross-HI tables were merged and mapped in 3 dimensions using antigenic cartography to quantify antigenic distances between antigens^42^. Antigenic distances between viruses were calculated in antigenic units (AU), in which 1 AU is equivalent to a 2-fold loss in HI cross-reactivity. Antigenic distances were plotted using GraphPad Prism 9 (GraphPad, San Diego, CA).

### In vivo pathogenesis and transmission study

The A/swine/North Carolina/A02751333/2022 (NC/22) isolate was propagated and titrated in Madin-Darby canine kidney (MDCK) cells using Opti-MEM (Life Technologies, Waltham, MA) containing antibiotics/antimycotics and 1 µg/ml of tosylsulfonyl phenylalanyl chloromethyl ketone (TPCK)-trypsin (Worthington Biochemical Corp., Lakewood, NJ) and clarified virus from infected cell culture was used for challenge. Back-titration of challenge inoculum was done to confirm the desired titer of 3.16 × 10^5^ 50% tissue culture infectious dose (TCID_50_)/ ml. Three-week-old piglets were obtained from a commercial source free of IAV and prophylactically treated with a dose of ceftiofur crystalline free acid and tulathromycin (Zoetis Animal Health, Florham Park, NJ) prior to transport. After arrival, pigs were screened for IAV antibodies by a commercial nucleoprotein ELISA kit (Swine Influenza Virus Antibody Test, IDEXX, Westbrook, ME), subcutaneously microchipped for body temperature monitoring (Destron Fearing™, Dallas, TX) and randomly divided into three groups: negative control (*n* = 5), primary challenge (*n* = 10), and indirect contact (*n* = 5). All groups were housed separately in biosafety level 2 containment rooms and cared for in compliance with the Institutional Animal Care and Use Committee of the National Animal Disease Center. On day 0, primary pigs were inoculated by intratracheal (2 ml) and intranasal (1 ml) routes under sedation using an intramuscular injection of ketamine (8 mg/kg of body weight; Phoenix, St. Joseph, MO), xylazine (4 mg/kg; Lloyd Inc., Shenandoah, IA), and Telazol (6 mg/kg; Zoetis Animal Health, Florham Park, NJ) cocktail. Nasal swabs (NS) from negative controls and primary challenged pigs were collected daily until 5 days post-inoculation (dpi), in media containing 2 ml minimum-essential-medium (MEM) supplemented with 1:1000 of 1 µg/ml TPCK Trypsin. On 2 dpi, five indirect naïve contact piglets were introduced into the challenge containment room and kept in a separate raised deck. Routine nasal swab collection of the contact pigs began on day 0 post-contact (dpc) and continued on 1, 2, 3, 4, 5, 7 and 9 dpc. At 5 dpi, the negative control and challenge groups were humanely euthanized with a lethal dose of pentobarbital (Fatal Plus; Vortech Pharmaceuticals, Dearborn, MI). At necropsy, blood and nasal swabs were collected and lungs were aseptically removed and lavaged with 50 ml of MEM containing 1% bovine serum albumin (BSA) to obtain bronchoalveolar lavage fluids (BALF). The percentage of the lung affected with purple-red consolidation typical of IAV in swine was visually estimated at 5 dpi as previously described^43^. Tissue samples from the trachea and right middle or affected lung lobe were fixed in 10% buffered formalin for histopathologic examination. Tissues were processed by routine histopathologic procedures, slides stained with hematoxylin and eosin (H&E), and lesions scored by a veterinary pathologist^43^. Serum samples were obtained from the contact pigs at 13 dpc. Lung lesion scores, log10-transformed virus titers, and log_2_-transformed HI reciprocal titers were analyzed using *t*-test, with a *p* value of <0.05 considered significant (GraphPad Prism software version 9; San Diego, CA).

Nasal swab samples were filtered with a 0.2-micron syringe filter and plated onto 24-well plates containing 80% confluent MDCK cells and cultured at 37 °C for 48 h, using media only rows as the negative control. Plates were fixed and stained for immunocytochemistry (ICC) as previously described^44^. BALF samples were streaked (100 µl) on Casmin (NAD-enriched) and blood agar plates to evaluate bacterial growth. BALF was also tested for *Mycoplasma hyopneumoniae*, porcine reproductive and respiratory syndrome virus and porcine circovirus type 2 according to ISU VDL SOP. Virus isolation-positive nasal swabs and BALF samples were titrated in 10-fold serial triplicate dilutions on 96-well plates of MDCK cells. At 48 h, plates were fixed and stained^44^. Titers were calculated for each sample as 50% tissue culture infectious dose (TCID_50_) per ml and transformed to log_10_ for comparison purposes. RT-rtPCR was performed to confirm virus isolation detection in nasal swab and BALF samples (VetMAX™-Gold SIV Detection Kit, Thermo Fisher Scientific, MA).

## Results

### Multiple detections of human-origin H3 influenza A virus in swine

Nineteen human-to-swine transmission events of seasonal H3 IAV were detected by molecular or virologic methods in swine samples submitted to the ISU VDL between November 7, 2022 to November 28, 2023 (Table 1). Fifteen IAV were sequenced from oral fluids and four from nasal swabs; virus isolates were obtained from two nasal swab samples, A/swine/North Carolina/A02751333/2022 and A/swine/North Carolina/A02758610/2023. Samples were collected from nine U.S. production premises: 3 from Colorado, Illinois, Indiana, 2 from Missouri, 7 from North Carolina, Ohio, and Pennsylvania. Additionally, 1 case was collected from Mexico and 2 cases from Chile.

**Table 1:** List of USDA barcodes, received date, state, sample type, and genetic lineage of the HA and NA for each reverse zoonotic H3 strain

USDA or ISU Code	Received date	State	Sample type	Mixed clade	HA clade	NA clade	WGS
A02751184	Nov 07, 2022	North Carolina	Oral fluid	H3-N1/N2	3C.2a1b.2a.2b	–	
A02751292	Dec 01, 2022	Ohio	Oral fluid		3C.2a1b.2a.2a.1	–	
A02751329	Dec 05, 2022	North Carolina	Oral fluid		3C.2a1b.2a.2b	–	
A02751330	Dec 07, 2022	North Carolina	Oral fluid		3C.2a1b.2a.2b	–	
A02751333^a^	Dec 13, 2022	North Carolina	Nasal swab		3C.2a1b.2a.2b	Human N2	Yes
A02751500	Jan 10, 2023	North Carolina	Oral fluid		3C.2a1b.2a.2b	–	
A02524408	Jan 13, 2023	Missouri	Oral fluid		3C.2a1b.2a.2a.1	Human N2	
A02758600	Jan 20, 2023	Pennsylvania	Oral fluid		3C.2a1b.2a.2b	Human N2	Yes
A02758601	Jan 26, 2023	Illinois	Oral fluid		3C.2a1b.2a.2a.1	N2.2002B	
A02758605	Feb 07, 2023	Missouri	Oral fluid	H1/H3-N1 (1A.3.3.3)	3C.2a1b.2a.2a.1	Human N2/N1.Classical	Yes
A02758604	Feb 21, 2023	Indiana	Oral fluid	H1/H3-N2 (1B.2.2.1)	3C.2a1b.2a.2b	N2.1998	Yes
A02758606	Apr 26, 2023	Colorado	Oral fluid	H1/H3-N2(1A.3.3.2)	3C.2a1b.2a.2b	Human N2	Yes
A02758607	May 01, 2023	North Carolina	Oral fluid		3C.2a1b.2a.2a.1	Human N2	Yes
A02758608	May 10, 2023	Colorado	Oral fluid		3C.2a1b.2a.2a.1	Human N2	
A02758609	May 11, 2023	Colorado	Oral fluid		3C.2a1b.2a.2a.1	Human N2	
A02758610^a^	May 22, 2023	North Carolina	Nasal swab		3C.2a1b.2a.2b	Human N2	Yes
ISU78274	Aug 18, 2023	Mexico	Oral fluid		3C.2a1b.2a.2b		
ISU97104	Oct 19, 2023	Chile	Nasal swab	H1/H3-N2 (Other-Human-1B.2/1A.3.3.2)	3C.2a1b.2a.2b		
ISU11011	Nov28. 2023	Chile	Nasal swab	H1/H3-N2 (Other-Human-1B.2)	3C.2a1b.2a.2a.1		

^a^Denotes viral isolate obtained.

Maximum-likelihood phylogenetic inference indicated that all HA sequences clustered within the human 3C.2a1b.2a lineage, with sequences falling into the 3C.2a1b.2a.2b and 3C.2a1b.2a.2a.1 clades (Table 1 and Fig. 1), herein referred to as clades 2b and 2a.1, respectively. The HA genes collected from swine were placed in multiple locations across the phylogeny, nested within clades of human seasonal HA genes indicating that multiple human-to-swine spillovers occurred during this human influenza season (Fig. 1). Three cases were detected as co-infections with endemic swine lineage viruses. Based on WGS of the HA and NA gene from clinical samples, cases A02758604–A02758606 were co-detected with H1 subtype IAV: H1.1A.3.3.3 (gamma), H1.1B.2.2.1 (delta1a), and H1.1A.3.3.2 (pdm09), respectively, with case A02758604 also containing an N1.Classical sequence. Pairwise differences between sequences derived from the clinical cases were calculated across the entire HA (1701nt), with a maximum difference of 42nt between all 19 sequences, 40nt differences among seasonal H3 clade 2b sequences, and 34nt among the seasonal H3 clade 2a.1 clade sequences (Supplementary Table 1).

There were eight notable clade-specific amino acid differences between the 2a.1 and 2b lineages. Using H3 numbering with the signal peptide trimmed, these amino acids labeled with the 2a.1 lineage first are E50K, G53D, F79V, G104D, I140K, S156H, R276K, and R503K in the HA2 (Supplementary Fig. 1). Six amino acid changes were found on the nine human NA, A238T, V263I, R315S, G346S, N463D, and S465N (Supplementary Fig. 2). Two antigenic motifs were present, distinct to each lineage. The 2a.1 lineage had a motif of STSNNK, and the 2b lineage motif was STHNNK. Notably, the only difference is at position 156, where 2a.1 had a serine and 2b had a histidine. The motifs found in the swine sequences matched the motifs of genetically similar human sequences.

Glycosylation was predicted at amino acid positions 7, 21, 37, 62, 132, 164, 245, and 284 for all U.S. swine-origin 2b clade sequences using H3 numbering, including the isolate A/swine/North Carolina/A02751333/2022 used in phenotypic characterization. ISU78274 and ISU97104 from Mexico and Chile respectively were predicted to have lost glycosylation at position 132 and 164, with strain ISU97104 potentially gaining glycosylation at position 125. Predicted glycosylation sites for the human-like 2a.1 clade HA additionally included position 44. Case A02751292 was an exception and lacked a predicted glycosylation at position 44, and case ISU110711 from Chile had an additional glycosylation at position 125.

Sanger sequencing for neuraminidase was performed on eleven cases, yielding eleven N2 subtype sequences and one N1 sequence from a co-detection case (Table 1). Nine sequences were human-origin N2, suggesting that the surface proteins of these viruses were still wholly human-origin (Fig. 2). The human-origin N2 sequences formed two clades on the tree, analogous to their paired H3 lineage. Two neuraminidases from A02758601 and A02758604 were related to endemic swine NA lineages N2.2002B and N2.1998 respectively. One sample, A02758605, contained an N1.Classical lineage NA, which was likely associated with the H1 1A.3.3.3 clade HA co-detected in the sample^45^. The sample type for the co-detections was oral fluid, a pooled population sample, thus no conclusion can be drawn about co-infection or reassortment between the viruses. The swine-origin N2.2002B lineage neuraminidase was compared to similar sequences on *ISU FLU*ture via BLAST and found to be genetically similar to NA sequences commonly paired with 1990.4.a H3 HA. The N2.1998 lineage sequence identified from A02758604 was genetically similar to NA found associated with 1990.1 H3 HA^1^.

### Mixed internal genes from co-detections

**Fig. 3:**
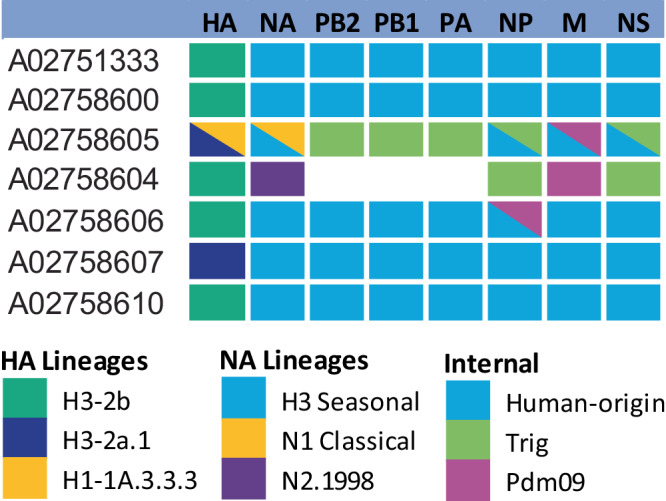
Genetic lineage constellations of whole genome sequences. Co-detections are marked via triangles. The polymerases of A02758604 were not able to be sequenced.

Whole genome sequencing (WGS) was performed on seven clinical samples and two isolates (Fig. 3). Four samples (A02751333, A02758600, A02758607, A02758610) yielded only human-origin gene segments. Sample A02758606 yielded 8 human-origin segments, and an additional pandemic 2009 lineage NP segment was detected. Sample A02758604 yielded a human seasonal H3 clade 2b HA, N2.1998 NA, TRIG NP and NS, and a pandemic M. No reliable length of sequence was recovered for any polymerase gene in this sample. Sample A02758605 yielded a mixture of human-origin and swine triple-reassortant origin internal genes, with both a human-origin and pandemic 2009 M protein. The WGS results on clinical samples indicated that the human-origin H3 viruses circulated concurrently in barns that also harbor endemic swine lineage IAV.

H3 was the predominant subtype detected in humans from 2010–2011 to 2012–2013, 2014–2015, 2016–2017 to 2017–2018, and 2021–2022 and 2022–2023 flu seasons. However, based on available CDC H3 detection data, there was no evident correlation between the prevalence of H3 in humans during an influenza season and the number of detected human-to-swine spillover cases (Pearson correlation = 0.13).

### Temporal reconstruction suggests swine-to-swine transmission

Temporal phylogenetic trees were inferred for U.S. sequences using Bayesian inference methods. All sequences included in the tree coalesced to a single common ancestor in late June of 2020 (95% HPD Jan 30, 2020–Nov 16, 2020). The mean rate of substitution was 3.99 × 10^−3^ substitutions/site/year (95% HPD 3.56 × 10^−3^ to 4.46 × 10^−3^ substitutions/site/year), which equates to approximately 6.79 substitutions/year across the 1701nt of the HA gene.

Bayesian inference inferred a mean of 8.27 host transitions over the span of the phylogeny (95% HPD 7–10), indicating between 7 to 10 independent human-to-swine spillover events occurred within the United States. The maximum clade consensus tree revealed geographic patterns between the sequences. Seven North Carolina sequences were in the human seasonal H3 2b clade, with six sequences forming a single group, potentially representing a swine-to-swine transmission chain (Supplementary Fig. 3a). A clade composed of sequences from North Carolina and Pennsylvania was also present in the human seasonal H3 2b clade, with the remaining events being single detections.

The largest human seasonal H3 clade 2a.1 group contained sequences from Colorado and a single gene sequence isolated in Missouri 4 months prior to the Colorado detections. The Colorado cases occurred across a 3-week span, and it was not clear whether this grouping of genes was derived from a single or multiple interspecies transmission events (Supplementary Fig. 3b). All other H3 2a.1 clades occurred as single events on the maximum-likelihood tree.

### Serologic evidence of human-origin H3 spillover into commercial swine herds

Pig serum samples from Mexico (farm A) and U.S. (farm B, C, D, and E) displayed HI positivity (HI ≥40) to a contemporary human seasonal H3N2 (A/Darwin/6/2021) and NC/22, but limited HI cross-reactivity to H1 human seasonal or other contemporary swine H3N2 representative strains of the 1990.4.a, 2010.1 and 2010.2 lineages. Farm A in Mexico had 75% of the serum samples HI positive to A/Darwin/6/2021 and NC/22. Farm B from the U.S. had 85% of serum samples HI positive for A/Darwin/6/2021 and 100% for NC/22. U.S. farm C had 60% of serum samples HI positive for A/Darwin/6/2021 and 70% for NC/22. U.S. farm D had 50% of serum samples HI positive for A/Darwin/6/2021 and 80% for NC/22. U.S. farm E had 100% of serum samples HI positive for A/Darwin/6/2021 and NC/22 (Table 2). These data confirmed that the H3N2 virus introduction was antigenically related to a human seasonal H3N2 (A/Darwin/6/2021).

**Table 2:** Serologic evidence of human to swine H3N2 transmission in breeding farms from Mexico and U.S.A

Farm	Country (state)	A/Darwin/6/2021 (H3N2 clade 2a.1)^a^	A/swine/North Carolina/A02751333/2022^a^	A/Hawaii/70/2019-like (H1N1 - HuVac)^a^	A/sw/Iowa/A02750897/2022 (1990.4.A)^a^	A/swine/Iowa/A02636454/2022 (2010.1)^a^	A/swine/Indiana/A02635878/2021 (2010.2)^a^	% Positives (positives/total samples)^b^	% Positives (positives/total samples)^c^
A	Mexico	50	65	10	10	16	10	75 (18/24)	75 (18/24)
B	US (MN)	51	72	10	10	11	11	85 (12/14)	100 (14/14)
C	US (MO)	40	46	10	10	10	10	60 (6/10)	70 (7/10)
D	US (NC)	32	70	10	10	28	10	50 (5/10)	80 (8/10)
E	US (WI)	98	139	10	10	13	10	100 (5/5)	100 (5/5)

^a^Geometric mean titers.

^b^Number of samples HI ≧ 40 against A/Darwin/6/2021.

^c^Number of samples HI ≧ 40 against A/swine/NC/A02751333/2022.

### Human-like swine isolate was antigenically distinct from contemporary swine IAV lineages

**Fig. 4:**
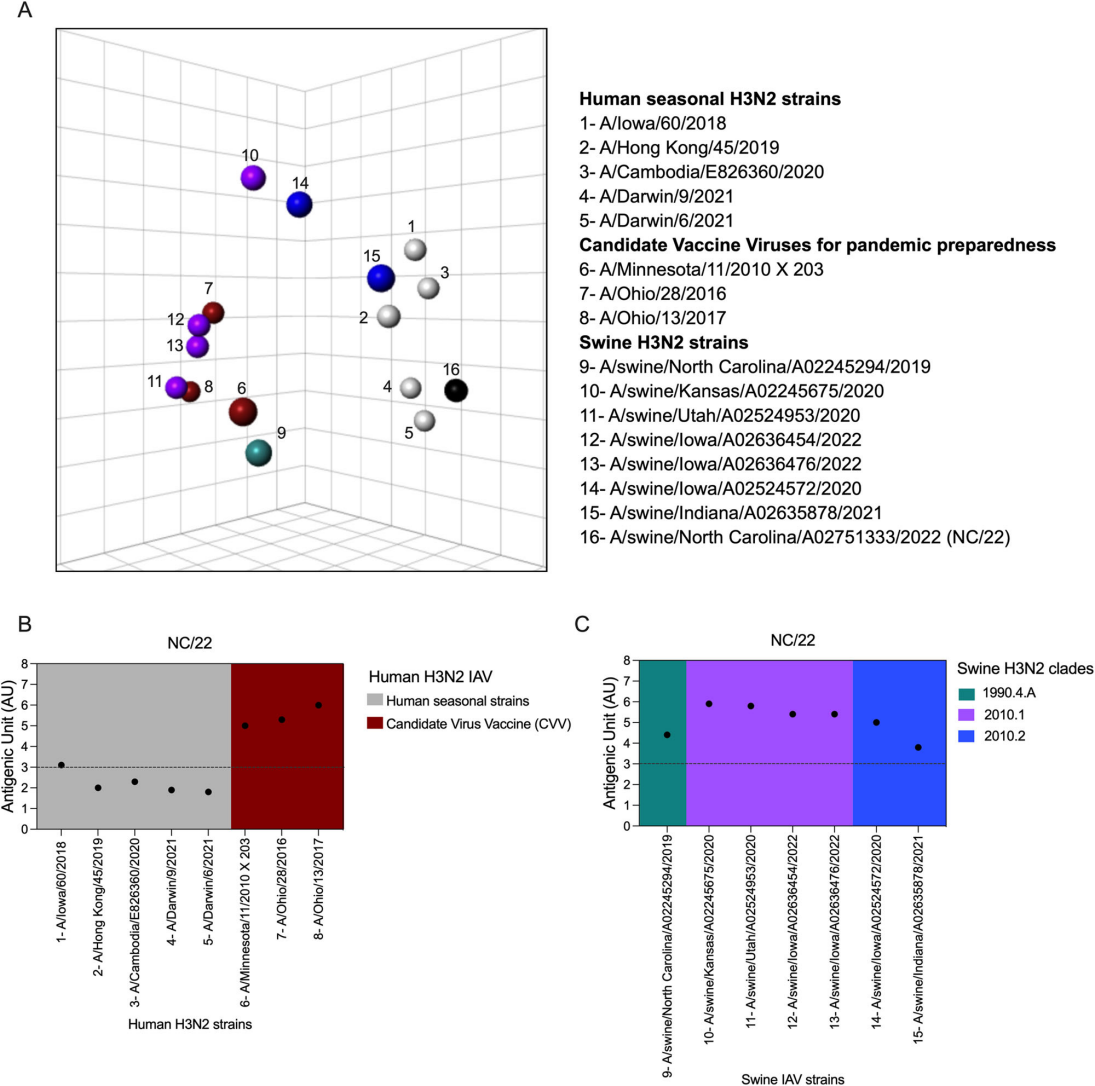
Antigenic characterization of NC/22 by ferret anti-sera. **A** Three-dimensional map showing antigenic relationships between swine NC/22 and human seasonal H3N2 vaccine strains, candidate vaccine viruses (CVV)s and contemporary swine H3N2 representative strains. Spheres represent each antigen in the map colored by lineage: human seasonal H3N2 vaccine strains (gray), CVV (dark red), 1990.4.A (turquoise), 2010.1 (purple) and 2010.2 (blue). Numbers in the map indicate strain name in the key. Each square of the background grid in the map corresponds to 1 antigenic unit (AU), which represents a two-fold loss in HI cross-reactivity. **B** Antigenic distances of human seasonal H3N2 strains and CVVs from NC/22. **C** Antigenic distances of swine 1990.4.A, 2010.1 and 2010.2 lineages from NC/22. The antigenic distance is reported in antigenic units (AU).

Antigenic relationships between A/swine/North Carolina/A02751333/2022 (NC/22 and human seasonal H3N2 strains, CVVs, and contemporary swine H3N2 strains are shown in the three-dimensional antigenic map (Fig. 4A and Supplementary Table 2). NC/22 (clade 2b) showed a closer antigenic similarity to the recent human seasonal H3N2 vaccine strains A/Darwin/9/2021 and A/Darwin/6/2021-like (clade 2a.1) with 1.8 and 1.9 AU, respectively. NC/22 retained antigenic similarity of <3 AU with other human seasonal H3N2 strains such as A/Hong Kong/45/2019 and A/Cambodia/E826360/2020. In contrast, NC/22 was 3.1 AU away from a human seasonal A/Iowa/60/2018 and more than 5.0 AU from the tested pandemic preparedness CVVs (Fig. 4B). The antigenic distances between NC/22 and swine H3N2 contemporary strains from 1990.4.a, 2010.1, and 2010.2 lineages showed a range of 3.8 to 6.0 AU (Fig. 4C). These results demonstrate that NC/22 is antigenically distinct from endemic swine H3N2 and antigenically similar to a human seasonal H3N2 strains of 2a.1.

### Pigs challenged with human-origin H3N2 demonstrated viral replication and shedding but no transmission to indirect contact pigs

Three-week-old pigs were challenged with A/swine/North Carolina/A02751333/2022 (NC/22) virus isolate to evaluate pathogenesis and transmission. At the 5 dpi necropsy, minimal to mild lesions typical of experimental IAV infection were observed, and there was no significant difference between the weighted average lung lesion percentages between the negative (non-challenged) group and the NC/22 challenged group of pigs. This was consistent with microscopic lung and tracheal lesion scores (Table 3). Virus shedding was detected in nasal swabs of all primary inoculated pigs with variable titers. Onset of nasal shedding was 1 dpi, and peak group shedding was recorded at 4 dpi. The NC/22 strain replicated at a mean titer of 4.67 log_10_ TCID_50_/ml in the lungs of inoculated pigs (Table 4). Despite sustained shedding from primary pigs, airborne transmission of NC/22 strain to indirect contact pigs was not detected. All nasal swabs from contact pigs throughout the study were negative for IAV by virus isolation and RT-rtPCR. HI assays confirmed a lack of airborne transmission with an absence of seroconversion at 13 dpc.

**Table 3:** Macroscopic, microscopic and IHC lung and trachea scores

	Lung			Trachea	
Group	Macroscopic (%)	Microscopic (0–22)	IHC (0–8)	Microscopic (0–8)	IHC (0–4)
Neg-control	0.0 ± 0.0	0.7 ± 0.4	0.7 ± 0.7	0.4 ± 0.2	0.2 ± 0.2
NC/22	0.8 ± 0.5	1.4 ± 0.5	2.1 ± 0.6	0.9 ± 0.3	0.7 ± 0.3

Values are means ± standard errors of the means (SEM). Microscopic lung lesion scores ranged from 0 to 22, IHC lung score ranged from 0 to 8. Microscopic trachea lesion scores ranged from 0 to 8 and IHC trachea score ranged from 0 to 4.

**Table 4:** Nasal shedding of primary pigs challenged with A/swine/North Carolina/A02751333/2022

Group	0 dpi	1 dpi	2 dpi	3 dpi	4 dpi	5 dpi	BALF 5 dpi
Neg-control	0.0 ± 0.0	0.0 ± 0.0	0.0 ± 0.0	0.0 ± 0.0	0.0 ± 0.0	0.0 ± 0.0	0.0 ± 0.0
NC/22	0.0 ± 0.0	2.5 ± 0.4 (9/10)	1.6 ± 0.5 (9/10)	2.9 ± 0.5 (10/10)	3.3 ± 0.4 (10/10)	3.7 ± 0.1 (10/10)	4.7 ± 0.4 (10/10)

Values are means of Log_10_ TCID_50_/ml ± standard errors of the means (number of positive/total of animals tested).

## Discussion

During the 2022–2023 human influenza season, repeated H3 reverse zoonotic spillovers from humans into swine were detected. While human-to-swine transmission events were detected in the past through routine sampling by the ISU VDL, the number of independent introductions and overall detections during a single human influenza season is the highest recorded to date. The detected viruses were from two phylogenetic clades estimated to have diverged in mid-2020 in humans, emphasizing that multiple spillover events occurred and that genetically diverse IAV were introduced into swine. HI titers detected against the human-like swine isolate from the human seasonal H3 2b clade and human seasonal H3 2a.1 clade virus indicate these strains were antigenically novel in swine and would further diversify the antigenic landscape if they became endemic. The spillover viruses in this study originated from the same H3 human seasonal lineage that seeded the H3.2010.1 and H3.2010.2 lineages circulating in swine from the previous decade. The lack of antibody cross-reactivity is reflective of the divergence of these lineages post species transitions and suggests the antigenic phenotype may be a major contributing component to the transmission dynamics between swine and humans. While an isolate was not obtained for the 2a.1 clade, the World Health Organization’s recommendation for vaccine composition in the northern hemisphere in 2023–2024 human serology experiments indicated that A/Darwin/6/2021-like (lineage 2a.1) post-vaccination virus neutralization geometric mean titers were not significantly reduced to either the 2b or 2a.1 clade^46^. This is suggestive that the vaccine candidate provides some protection to both 2b and 2a.1 lineages in humans and may indicate antigenic cross-reactivity in swine as well. This is supported by the HI titers in the sera from farms with antibodies to strains from both clades.

Eighteen cases from this study were identified from routine diagnostics requested by producers. Only one case, A02758610, was requested by the diagnostic lab to identify if human-origin virus was persisting. Although further sampling was not conducted, the serological evidence indicates that multiple swine in the field during the period of this study were exposed to human H3N2 IAV. An active sampling strategy of targeting farms with seropositivity could better characterize how these spillover viruses are affecting the genetic landscape of swine IAV. Previous work identified a linear correlation between H1N1pdm09 prevalence in humans and the number of human-to-swine spillovers^47^. A correlation between human seasonal H3 detections between humans and the number of interspecies transmission events was not observed in the current data. The lack of an observable correlation may be due to species barriers that typically minimize human-to-swine transmission of H3N2 when compared to the H1N1pdm09. The H1N1pdm09 lineage emerged in pigs and has some genetic components, including the HA gene, that have evolved in the swine host for nearly a century^48^. This contrasts with the human seasonal H3 influenza viruses that are circulated primarily in humans since 1968^3,4,49^. However, the unprecedented number of independent transmission events from humans into swine suggests the human seasonal H3N2 from 2022–2023 possessed a phenotype that may be more infectious to the swine host. The serological data indicated widespread exposure to human H3 viruses within multiple farm systems, thus there may be greater spread of human seasonal H3 viruses in the swine population that have not been detected through passive surveillance. Human seasonal clade 3C.2a1b.2a.2 viruses were noted for having altered glycosylation and sialic acid binding properties in humans^50^. This change in biochemical properties may have provided these H3N2 viruses a selective advantage in swine. Prior literature has noted the tendency for endemic swine IAV to be less glycosylated than human lineages^51^. The human seasonal 2b viruses were predicted to lack glycosylation at position 44 compared to 2a.1 H3 genes, and this subclade was associated with swine-to-swine transmission of the 2b lineage as observed in North Carolina. Notably, U.S. 2b strains had 8 predicted glycosylation sites while strains from Mexico and Chile had 6 and 7 predicted sites, respectively.

Despite the high numbers of human seasonal H3 detections in swine, it is unknown if any of the reverse-zoonotic H3 introductions will become endemic in the swine population. Indication of viral spread between production systems and state borders was not observed. Multiple co-detections with endemic swine IAV were found in this study, but there were no clear indication of reassortment between the human seasonal H3N2 spillover strains and endemic swine viruses that has previously been detected as a factor in the persistence of novel spillovers^2,5,14^. The sample type for the co-detections was oral fluid, a pooled population sample, thus no conclusion can be drawn regarding co-infection or reassortment between the viruses. We did not detect samples with single gene segment origins representing a reassortant IAV isolate. However, the occurrence of co-detections in 3 out of 16 cases ( ~ 19%) demonstrates these spillovers are occurring in production systems with active swine IAV infections, showing reassortment events are possible. The lack of sufficient samples and sequences from Mexico and Chile make it impossible to discern the spread in this region. Establishment of a new H3 clade in swine has the potential to further complicate vaccine-based control efforts by increasing viral diversity of endemic IAV circulating in swine as described in prior spillovers represented by the H3.2010.1 and H3.2010.2^2,5^.

A notable challenge of this study was the lack of virus isolates obtained. Antigenic characterization as well as sequencing of the internal genes is easier and often more successful to perform if a virus can be isolated from a sample and propagated to high titers. The majority of sample types diagnosed with human-like H3N2 were oral fluid, a minimally invasive sample to obtain from pigs and a common diagnostic specimen used for surveillance the RT-rtPCR cycle threshold (CT) values are typically higher, and successful virus isolation and sequencing is more difficult compared to other antemortem sample types such as a nasal swab. As oral fluids are a pooled sample, it is also difficult to determine if an IAV codetection is derived from a single virus or detection of individual IAV infection from multiple swine that contributed to the oral fluid sample. For future detections, cases of human-to-swine spillovers should be followed by epidemiological investigations with a request to the client for nasal swabs, nasal wipes, and other individual respiratory samples with the goal of obtaining isolates for further characterization. Our study also included serologic evidence that confirmed exposure to a virus that is antigenically similar to recent human H3 seasonal strains in commercial breeding herds where virologic identification of transmission was unsuccessful. This suggests additional spillover events and potential circulation of this human seasonal H3N2 virus clade in pig farms and regions is likely to have gone undetected or under-reported. Under-surveillance is concerning as prior human-to-swine spillovers have persisted for multiple years, reassorted to acquire endemic swine IAV genes, and then dramatically increasing in detection frequency so that the genetic and antigenic landscape of IAV in swine was shifted^5,52,53^.

Our in vivo study demonstrated that the human-origin H3N2 virus (NC/22 strain) carrying a complete human (H) origin internal gene constellation HHHHHH replicated efficiently in the upper and lower respiratory tract of directly inoculated pigs without causing significant lesions. This suggests pigs may be subclinical or exhibit mild clinical disease in farms with human seasonal H3N2 spillovers. Despite consistent nasal viral shedding from primary inoculated pigs, there was no aerosol transmission detected in indirect contact pigs, explaining the minimal evidence of onward transmission within and between production systems. We recognize that our study design only allowed for the assessment of airborne viral transmission and therefore our results might dismiss the ability of this virus to transmit in field conditions where direct nose-to-nose transmission occurs. However, we have previously established that well adapted swine H3N2 IAV are capable of transmitting efficiently in indirect contact experimental model^54,55^.

Internal gene constellations in combination with the surface glycoproteins HA and NA impact efficiency of virus replication, transmission pattern, and disease severity of IAV^2^. The 2022–2023 human viruses introduced into pigs may need further adaptation and/or reassortment with endemic swine IAV for efficient transmission and maintenance in the U.S. swine population. This study indicates an immediate potential threat to swine herd health with the introduction of an antigenically novel lineage of H3 IAV from humans. This study also underscores a reoccurring pattern of new introductions of human lineage IAV into swine, dictated by what is predominately circulating in humans^2,5,14,47^. The current best practice to prevent human-seasonal IAV introductions into swine herds are to adhere to stringent biosecurity protocols and to avoid letting ill humans interact with swine herds. Routine vaccination of swine has been shown to reduce viral shedding in swine^56,57^. Additionally, vaccination has been shown to reduce the chance of a reassortment event occurring, speculatively through the reduction in the number of days for a productive infection^58^. There is an inextricable link between IAV circulating in humans and swine. To break this pattern and mitigate future disease introduction additional focus will be needed to minimize spread of disease at the human-swine interface.

## Supplementary Information

Supplementary Information

## Data Availability

All sequences derived from United States cases in this study are listed by barcode in Table 1 and are available for download from Genbank. Foreign animal cases have not been submitted to Genbank but are available from the corresponding author on reasonable request.
