# Supplementary material for: Reverse zoonosis of the 2022–2023 human seasonal H3N2 detected in swine
Source: Npj Viruses. 2024 Aug 13;2:27. doi: 10.1038/s44298-024-00042-4 (PMC11721445; doi:10.1038/s44298-024-00042-4)

Supplemental Table 1. Number of pairwise nucleotide differences between each strain.

	A02758600|A/swine/Pennsylvania/7201/2023	A02751333|A/swine/North_Carolina/113947/2022	A02751330|A/swine/North_Carolina/113670/2022	A02751184|A/swine/North_Carolina/104698/2022	A02751500|A/swine/North_Carolina/3001/2023	A02758610|A/swine/North_Carolina/49318/2023	A02751329|A/swine/North_Carolina/111924/2022	A02758607|A/swine/North_Carolina/41356/2023	A02758604|A/swine/Indiana/17537/2023	ISU78274|A/swine/Mexico/78274/2023	ISU97104|A/swine/USA/97104/2023	A02758601|A/swine/Illinois/9465/2023	A02524408|A/swine/Missouri/5158/2023	A02758605|A/swine/Missouri/13860/2023	A02751292|A/swine/Ohio/112849/2022	A02758606|A/swine/Colorado/38854/2023	A02758608|A/swine/Colorado/42959/2023	A02758609|A/swine/Colorado/47025/2023	ISU110711|A/swine/Chile/110711/2023	
A02758600|A/swine/Pennsylvania/7201/2023		9	2	9	9	10	10	12	18	25	22	29	27	30	30	32	34	34	29	
A02751333|A/swine/North_Carolina/113947/2022	9		9	0	0	1	1	3	15	22	19	26	24	27	27	29	31	31	28	
A02751330|A/swine/North_Carolina/113670/2022	2	9		9	9	10	10	12	18	25	22	29	27	30	30	32	34	34	29	
A02751184|A/swine/North_Carolina/104698/2022	9	0	9		0	1	1	3	15	22	19	26	24	27	27	29	31	31	28	
A02751500|A/swine/North_Carolina/3001/2023	9	0	9	0		1	1	3	15	22	19	26	24	27	27	29	31	31	28	
A02758610|A/swine/North_Carolina/49318/2023	10	1	10	1	1		2	2	16	23	20	27	25	28	28	30	32	32	29	**2b**
A02751329|A/swine/North_Carolina/111924/2022	10	1	10	1	1	2		4	16	23	20	27	25	28	28	30	32	32	29	
A02758607|A/swine/North_Carolina/41356/2023	12	3	12	3	3	2	4		18	25	22	29	27	30	30	32	34	34	31	
A02758604|A/swine/Indiana/17537/2023	18	15	18	15	15	16	16	18		21	18	31	29	32	30	34	36	36	33	
ISU78274|A/swine/Mexico/78274/2023	25	22	25	22	22	23	23	25	21		17	37	35	38	36	40	42	42	39	
ISU97104|A/swine/USA/97104/2023	22	19	22	19	19	20	20	22	18	17		35	33	36	34	38	40	40	37	
A02758601|A/swine/Illinois/9465/2023	29	26	29	26	26	27	27	29	31	37	35		4	5	15	9	12	11	8	
A02524408|A/swine/Missouri/5158/2023	27	24	27	24	24	25	25	27	29	35	33	4		5	13	7	10	9	6	
A02758605|A/swine/Missouri/13860/2023	30	27	30	27	27	28	28	30	32	38	36	5	5		16	10	13	12	9	
A02751292|A/swine/Ohio/112849/2022	30	27	30	27	27	28	28	30	30	36	34	15	13	16		18	21	20	17	**2a.1**
A02758606|A/swine/Colorado/38854/2023	32	29	32	29	29	30	30	32	34	40	38	9	7	10	18		3	2	11	
A02758608|A/swine/Colorado/42959/2023	34	31	34	31	31	32	32	34	36	42	40	12	10	13	21	3		3	14	
A02758609|A/swine/Colorado/47025/2023	34	31	34	31	31	32	32	34	36	42	40	11	9	12	20	2	3		13	
ISU110711|A/swine/Chile/110711/2023	29	28	29	28	28	29	29	31	33	39	37	8	6	9	17	11	14	13		
						**2b**									**2a.1**					

Supplemental Table 2. Hemagglutination inhibition titers against homologous and heterologous strains merged and extracted from the antigenic map.

			Antisera																
No	Antigen name (H3N2)	Antigen passage	A/Iowa/60/2018	A/Iowa/60/2018	A/Hong Kong/45/2019	A/Cambodia/E826360/2020-F1	A/Cambodia/E826360/2020-F25	A/Darwin/9/2021	A/Darwin/6/2021-F5	A/Darwin/6/2021-F6	A/Minnesota/11/2010X203	A/Ohio/28/2016	A/Ohio/13/2017	A/swine/North Carolina/A02245294/2019-F18	A/swine/North Carolina/A02245294/2019-F32	A/swine/Utah/A02524953/2020-F19	A/swine/Utah/A02524953/2020-F20	A/swine/Iowa/A02524572/2020-F842	A/swine/Iowa/A02524572/2020-F843
1	A/Iowa/60/2018	MDCK2	320	320	*	80	80	*	40	40	<10	*	<10	<10	<10	<10	10	80	20
2	A/Hong Kong/45/2019	MDCK2	80	80	127	*	*	*	*	*	<10	<40	*	*	*	*	*	*	*
3	A/Cambodia/E826360/2020	MDCK2	160	160	*	160	101	*	40	80	<10	<10	<10	<10	<10	10	10	40	10
4	A/Darwin/9/2021	MDCK2	*	*	*	*	*	1280	320	640	<10	*	10	10	10	10	10	*	*
5	A/Darwin/6/2021	MDCK2	10	10	*	20	40	320	269	538	<10	10	<10	10	<10	20	20	20	10
6	A/Minnesota/11/2010X203	MDCK2	<40	<40	<10	<10	<10	20	80	10	861	<40	<40	160	80	40	80	20	40
7	A/Ohio/28/2016	MDCK2	13	13	<20	40	40	*	40	20	<40	2229	320	40	40	160	320	*	*
8	A/Ohio/13/2017	MDCK2	10	10	*	<10	<20	20	<40	<20	<10	80	1076	80	20	160	160	20	10
9	A/swine/North Carolina/A02245294/2019	MDCK2	<20	<20	<10	20	40	40	40	10	57	16	<40	905	1076	20	40	10	10
10	A/swine/Kansas/A02245675/2020	MDCK2	20	20	<10	*	*	*	*	*	10	40	*	*	*	*	*	*	*
11	A/swine/Utah/A02524953/2020	MDCK2	<10	<10	<10	<10	10	20	20	10	10	40	160	40	40	538	640	10	10
12	A/swine/Iowa/A02636454/2022	MDCK2	*	*	*	10	20	10	40	<10	10	40	160	40	20	320	320	*	*
13	A/swine/Iowa/A02636476/2022	MDCK2	*	*	*	10	40	*	40	10	10	160	160	80	40	640	640	*	*
14	A/swine/Iowa/A02524572/2020	MDCK2	40	40	<10	10	10	*	20	<10	20	20	20	20	20	10	20	640	320

15	A/swine/Indiana/A02635878/2021	MDCK2	10	10	20	10	20	20	20	<10	14	10	<10	20	20	20	40	*	*
16	A/swine/North Carolina/A02751333/2022	MDCK2	20	20	*	20	20	320	113	80	<10	*	<40	14	14	<20	<40	20	10

Key

(grey)	Homologous HI titer
*	Not tested (Merged Table)

MDCK=Madin-Darby Canine Kidney cells

Supplemental figure 1. Visualization of the HA alignment between swine-origin sequences collected in this study. Clade defining mutations have been annotated at their position.

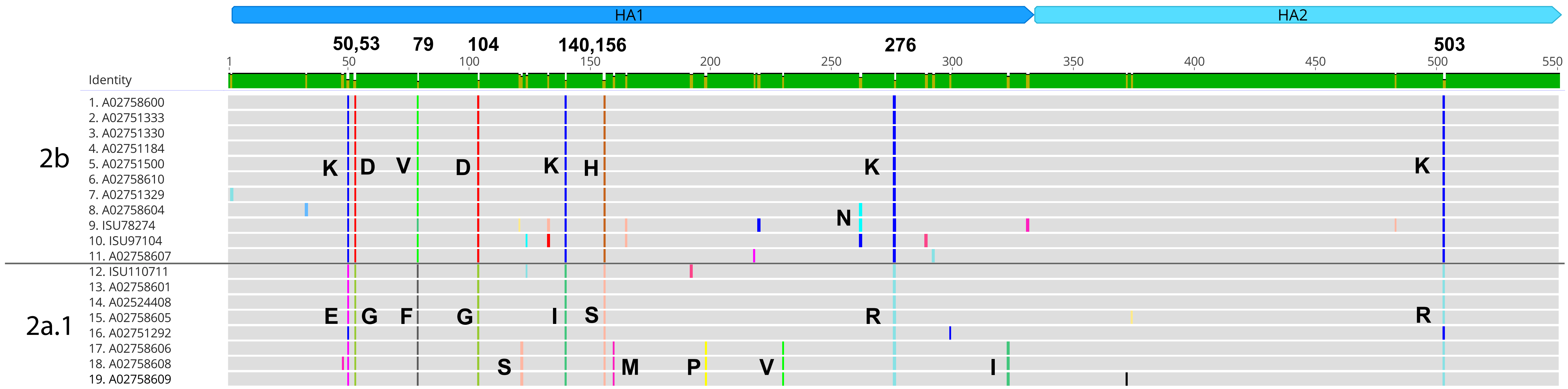

Supplemental figure 2. Visualization of the NA alignment between swine-origin sequences collected in this study. Clade defining mutations have been annotated at their position.

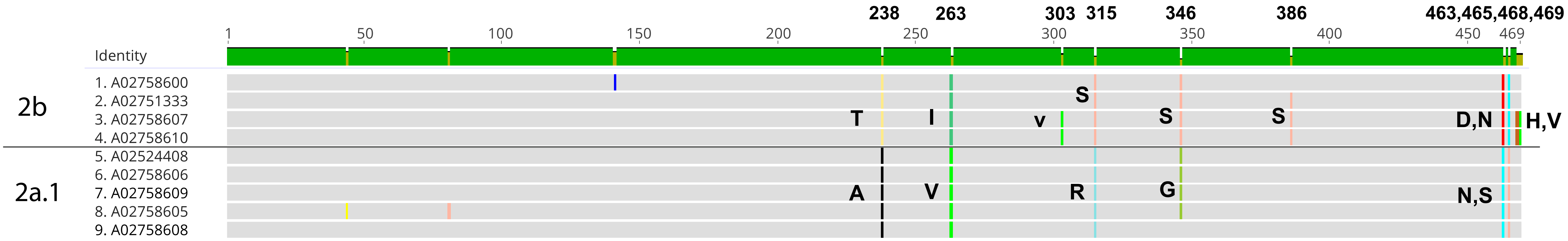

Supplemental figure 3. A time-scaled phylogeny for subtrees of A) 2b and B) 2a.1.

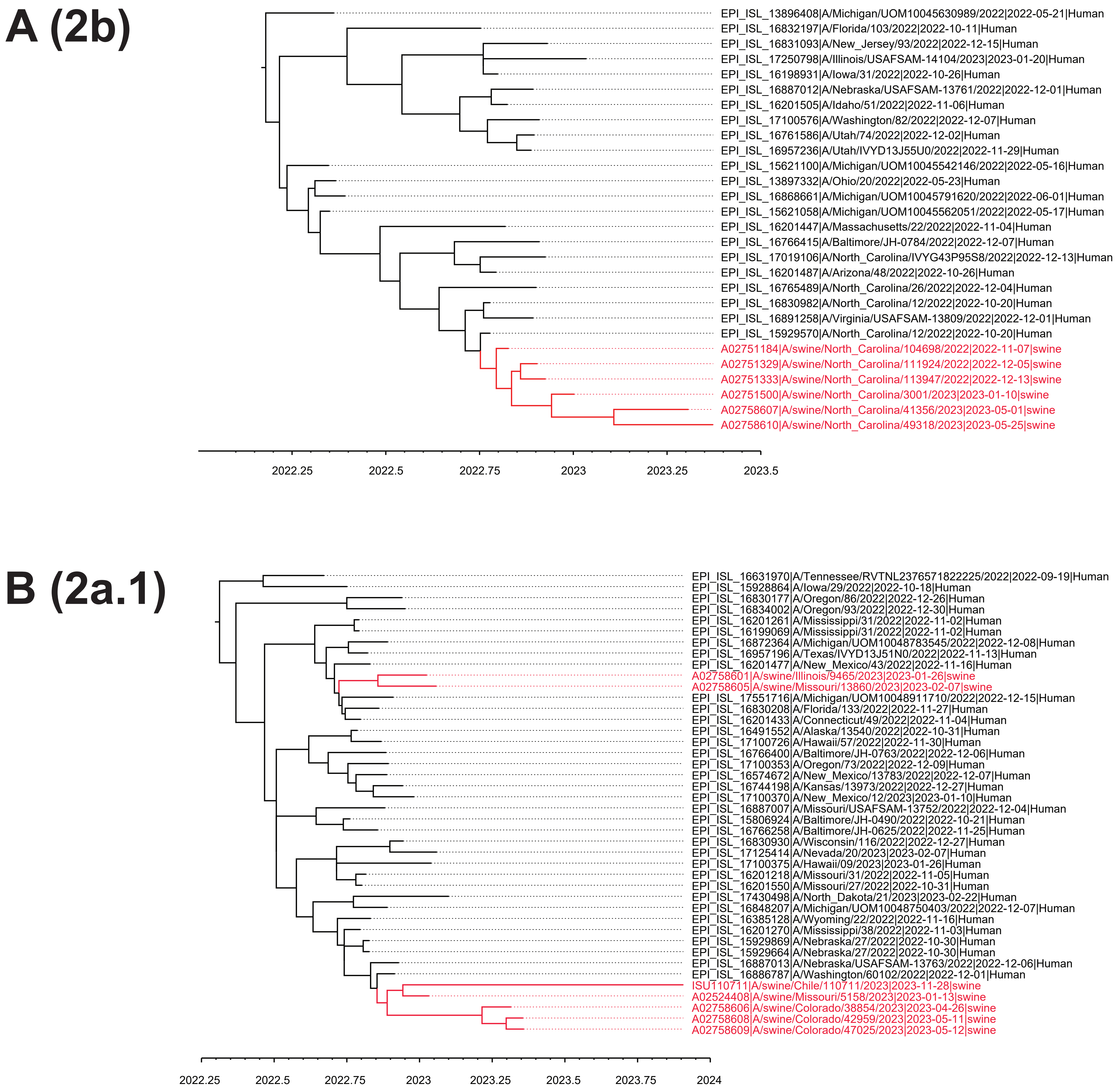

Supplement: Supplementary file 1 — Supplementary Information [file 44298_2024_42_MOESM1_ESM.pdf]
